# Incidence and risk of pancreatic cancer in patients with acute or chronic pancreatitis: a population-based cohort study

**DOI:** 10.1038/s41598-023-45382-y

**Published:** 2023-11-02

**Authors:** Seon Mee Park, Ki Bae Kim, Joung-Ho Han, Nayoung Kim, Tae Uk Kang, Heather Swan, Hyun Jung Kim

**Affiliations:** 1 Department of Internal Medicine, Chungbuk National University College of Medicine, Cheongju, Republic of Korea; 2 Department of Internal Medicine, Chungbuk National University Hospital, Cheongju, Republic of Korea; 3 Department of Internal Medicine, Seoul National University College of Medicine, Seoul, Republic of Korea; 4 Department of Internal Medicine, Seoul National University Bundang Hospital, Seungnam, Republic of Korea; 5 Health and Wellness College, Sungshin Women’s University, Seoul, Republic of Korea; 6 Department of Preventive Medicine, Korea University College of Medicine, 126-1, 5-ga, Inchon-ro, Seoul, 136-705 Republic of Korea; 7 Department of Public Health, Graduate School, Korea University, Seoul, Republic of Korea

**Keywords:** Gastroenterology, Oncology

## Abstract

We aimed to evaluate the incidence and risk of pancreatic cancer (PC) in pancreatitis. We identified patients with acute pancreatitis (AP) (n = 225,811, 50.0%) and chronic pancreatitis (CP) (n = 225,685, 50.0%) from Korean population-based data and matched them with age- and sex-matched controls (n = 4,514,960). We analyzed the incidence and adjusted hazard ratios (aHRs) of PC among patients followed for more than 2 years or 5 years, and assessed risk changes over time in single episode of AP (SAP), recurrent AP (RAP), CP with AP, and CP without AP groups. We also performed subgroup analysis for both sexes. The incidences (per 10^4^ person-years) and risks (aHR) of PC were higher in the RAP (12.69, 5.00) or CP with AP (12.12, 5.74) groups compared to the SAP (2.31, 1.32) or CP without AP (2.28, 1.57) groups. The risks of PC decreased over time, however, the risk of PC remained elevated in the RAP and CP with AP groups for more than 8 years. Females with RAP, SAP, and CP with AP had higher risks of PC than males. The risk of PC is higher and persists for longer duration in patients with RAP and CP with AP compared to those with SAP or CP without AP.

## Introduction

Chronic pancreatitis (CP) is a chronic inflammatory disease of the pancreas and a well-established risk factor of pancreatic cancer (PC), which is the leading cause of death in these patients^1^. As a result, surveillance for early detection of PC has been suggested, although clear screening guidelines do not exist for CP patients^2^.

The risk of PC in CP patients has been reported with varying results across different study populations. Cumulative incidences of PC among newly diagnosed CP patients have been reported to range from 0.24 to 5.7%^3–6^. The risks of PC among CP patients are widely reported fold increases ranging from 2.2 to 16.0 compared to controls^6–10^, largely due to differences in study populations.

The association between acute pancreatitis (AP) and PC has been a topic of debate^2^. Large cohort study has reported that both AP and CP increase the risk of PC^11^. In that study, the risk of PC in AP is associated with the numbers of recurrences rather than the etiology of pancreatitis^11^.

Pancreatitis manifests as different stages within the same disease spectrum, including single episode of acute pancreatitis (SAP), recurrent AP (RAP), early CP, established CP and end stage CP^12^. Although RAP and CP differ in terms of pancreatic morphology and/or histology, a subset of RAP patient may show histological evidence of CP. However, only a quarter to half of CP patients have a prior history of AP^13^. The clinical manifestations of CP also differ depending on presence or absence of previous AP^13^. CP patients with AP or RAP have higher rates of active smoking and alcohol-related complications compared to those without AP or SAP^12^. Patients who simultaneously smoke and drink alcohol have an increased frequency of inflammatory complications compared to those with a single etiological risk factor^14,15^. Prolonged inflammation in the pancreas plays a significant role in the initiation and development of PC^16^. Based on these considerations, our hypothesis is that CP patients with AP or RAP may have a higher risk of PC compared to those without AP or SAP.

In this study, we aimed to evaluate the risks of PC among CP patients with or without AP and AP patients with or without recurrence. We adjusted for other risk factors associated with PC, such as sex, age, smoking, drinking, obesity, diabetes, physical inactivity, and high cholesterol level. Additionally, we accounted for gallstone diseases, alcoholic liver diseases, and hepatic fibrosis or cirrhosis as confounding factors^17^.

## Results

### Patient characteristics

Table 1 provided the characteristics of patients with AP or CP and control group. The male-to-female ratio was 56.1:43.9, and the mean age was 56.8 years. Patients with pancreatitis exhibited higher rates of all risk factors associated with PC compared to the control group (SD ≥ 0.1 for each parameter), except for cholesterol level and physical activity, which did not differ significantly between the two groups. Patients with RAP and CP with AP demonstrated similar characteristics. They were predominantly males and had higher rates of hepatic fibrosis/cirrhosis, alcoholic liver disease, gallstone diseases, hepatic dysfunction, diabetes, alcohol consumption, and smoking compared to other groups. The age at initial diagnosis of AP in the CP with AP group was slightly older than that in the CP without AP group (mean age, 57.3 years vs. 55.8 years).

**Table 1:** Characteristics of patients with acute or chronic pancreatitis and controls.

	Control		Pancreatitis										
			Total		SD	SAP		RAP		CP with AP		CP without AP	
N, %	4,514,960	100	451,496	100		215,765	47.8	10,046	2.2	43,350	0.9	182,335	40.4
Sex													
Male	2,532,180	56.1	253,218	56.1	0.0	118,559	54.9	6299	62.7	28,894	66.7	99,466	54.6
Female	1,982,780	43.9	198,278	43.9		97,206	45.1	3747	37.3	14,456	33.3	82,869	45.4
Ages													
Mean, SD	56.8	14.6	56.8	14.6		57.5	15.0	57.9	14.4	57.3	13.1	55.8	14.1
Hepatic fibrosis/cirrhosis													
No	4,416,857	97.8	417,118	92.4	0.3	200,720	93.0	8774	87.3	38,217	88.2	169,407	92.9
Yes	98,103	2.1	34,378	7.6		15,045	7.0	1272	12.7	5133	11.8	12,928	7.1
Alcoholic liver disease													
No	4,133,035	91.5	367,254	81.3	0.3	179,211	83.1	6814	67.8	29,646	68.4	151,583	83.1
Yes	381,925	8.4	84,242	18.7		36,554	16.9	3232	32.2	13,704	31.6	30,752	16.9
Gallstones													
No	4,314,229	95.6	373,304	82.7	0.4	173,648	80.5	7800	77.6	33,434	77.1	158,422	86.9
Yes	200,731	4.4	78,192	17.4		42,117	19.5	2246	22.3	9916	22.9	23,913	13.2
ALT (U/L)													
< 40	4,030,496	89.3	384,172	85.1	0.1	184,261	85.4	8029	79.9	34,548	79.7	157,334	86.3
≥ 41	482,194	10.7	67,062	14.9		31,364	14.5	2009	20.0	8779	20.3	24,910	13.7
Missing	2270	0.1	262	0.1		140.0	0.1	8.0	0.1	23.0	0.1	91.0	0.0
GGT (U/L)													
< 76	3,814,110	84.5	344,296	76.3	0.2	165,122	76.5	6495	64.7	28,184	65.0	144,495	79.2
≥ 76	699,254	15.5	106,998	23.7		50,530	23.4	3547	35.3	15,147	34.9	37,774	20.7
Missing	1596	0.0	202	0.0		113	0.1	4	0.0	19	0.0	66	0.0
BMI (Kg/m^2^)													
< 18.5	145,080	3.2	18,363	4.1	0.1	8519	3.9	400	4.0	1892	4.4	7552	4.1
18.5–24.9	2,804,428	62.1	276,791	61.3		129,380	60.0	5978	59.5	26,594	61.3	114,839	63.0
25.0–29.9	1,404,842	31.1	137,497	30.5		67,836	31.4	3173	31.6	13,133	30.3	53,355	29.3
≥ 30	160,610	3.6	18,845	4.2		10,030	4.6	495	4.9	1731	4.0	6589	3.6
FPG (mg/dL)													
< 110	3,657,263	81.0	349,586	77.4	0.1	166,752.0	77.3	7454	74.2	32,142	74.1	143,238	78.6
110–125	471,938	10.5	51,795	11.5		24,900.0	11.5	1291	12.9	5672	13.1	19,932	10.9
≥ 126	384,181	8.5	49,923	11.1		24,003.0	11.1	1297	12.9	5519	12.7	19,104	10.5
Missing	1578	0.0	192	0.0		110.0	0.1	4	0.0	17	0.0	61	0.0
Cholesterol (mg/dL)													
< 200	2,564,256	56.8	264,911	58.7	0.0	125,994	58.4	5735	57.1	25,192	58.1	107,990	59.2
200–239	1,401,953	31.1	132,225	29.3		63,425	29.4	2958	29.4	12,633	29.1	53,209	29.2
≥ 240	546,524	12.1	54,110	12.0		26,215	12.1	1345	13.4	5503	12.7	21,047	11.5
Missing	2227	0.0	250	0.1		131	0.1	8	0.1	22	0.1	89	0.0
Smoking (pack-year)													
None	2,896,207	64.1	277,035	61.4	0.1	133,110	61.7	5669	56.4	22,837	52.7	115,419	63.3
< 10	423,478	9.4	41,982	9.3		20,679	9.6	885	8.8	3927	9.1	16,491	9.0
11–20	476,184	10.5	49,221	10.9		23,320	10.8	1281	12.8	5944	13.7	18,676	10.2
21–30	311,992	6.9	34,852	7.7		16,303	7.6	943	9.4	4456	10.3	13,150	7.2
31–40	176,425	3.9	20,648	4.6		9597	4.4	530	5.3	2685	6.2	7836	4.3
≥ 40	176,142	3.9	21,923	4.9		10,209	4.7	573	5.7	2825	6.5	8316	4.6
Missing	54,532	1.2	5835	1.3		2547	1.2	165	1.6	676	1.6	2447	1.3
Alcohol (drinks/week)													
None	2,300,662	51.0	225,940	50.0	0.1	112,014	51.9	4511	44.9	18,416	42.5	90,999	49.9
< One/month	981,054	21.7	89,814	19.9		43,476	20.1	1688	16.8	7813	18.0	36,837	20.2
< One/week	288,408	6.4	27,642	6.1		11,311	5.2	832	8.3	3436	7.9	12,063	6.6
≥ One/week	622,561	13.8	76,975	17.0		35,710	16.6	2202	21.9	10,454	24.1	28,609	15.7
Missing	322,275	7.1	31,125	6.9		13,254	6.1	813	8.1	3231	7.5	13,827	7.6
Physical activity													
None	2,330,298	51.6	240,277	53.2	0.0	118,223	54.8	5625	56.0	23,189	53.5	93,240	51.1
Light-moderate	1,204,603	26.7	116,288	25.8		53,948	25.0	2313	23.0	10,985	25.3	49,042	26.9
Vigorous	923,023	20.4	89,138	19.7		41,146	19.1	1935	19.3	8553	19.7	37,504	20.6
Missing	57,036	1.3	5793	1.3		2448	1.1	173	1.7	623	1.4	2549	1.4

*SAP* single episode of acute pancreatitis, *RAP* recurrent acute pancreatitis, *CP* chronic pancreatitis, *SD* standardized mean difference, *ALT* alanine transaminase, *GGT* gamma-glutamyl transferase, *BMI* body mass index, *FPG* fasting plasma glucose.

Table 2 presented the clinical characteristics of PC cases in the pancreatitis and control groups. In comparison to the control group, PC cases in the pancreatitis group were younger (mean ages, 69.0 vs. 65.5), had a higher frequency of gallstones (14.3% vs. 21.4%), and had a higher prevalence of smoking and alcohol consumption.

**Table 2:** Characteristics of pancreatic cancer cases in pancreatitis and control groups.

	Pancreatic cancer				
	Controls		Pancreatitis		SD
N, %	5901	100	12,780	100	
Sex					
Male	3629	61.5	7641	59.8	0.03
Female	2272	38.5	5139	40.2	
Ages					
Mean, SD	69.0	9.3	65.5	10.7	0.35
Hepatic fibrosis/cirrhosis					
No	5602	94.93	12,062	94.38	0.03
Yes	299	5.07	718	12.17	
Alcoholic liver disease					
No	5260	89.14	11,045	86.42	0.09
Yes	641	10.9	1735	13.6	
Gallstones					
No	5059	85.7	10,039	78.6	0.22
Yes	842	14.3	2741	21.4	
ALT (U/L)					
< 40	5310	90.0	11,271	88.2	0.06
41+	584	9.9	1497	11.7	
Missing	7	0.1	12	0.1	
GGT (U/L)					
< 76	4911	83.2	10,236	80.2	0.08
76+	984	16.7	2537	19.8	
Missing	6	0.1	7	0.0	
BMI (Kg/m^2^)					
< 18.5	143	2.4	313	2.5	0.02
18.5–24.9	3557	60.3	7711	60.3	
25.0–29.9	2004	34.0	4278	33.5	
30+	197	3.3	478	3.7	
FPG (mg/dL)					
< 110	4223	71.6	9186	71.9	0.02
110–125	842	14.3	1791	14.0	
126+	830	14.1	1796	14.0	
Missing	6	0.1	7	0.1	
Cholesterol (mg/dL)					
< 200	3305	56.0	7144	55.9	0.01
200–239	1833	31.1	3969	31.1	
≥ 240	756	12.8	1656	12.9	
Missing	7	0.1	11	0.1	
Smoking (pack-year)					
None	3856	65.3	7701	60.3	0.12
< 10	293	5.0	738	5.8	
11–20	551	9.3	1308	10.2	
21–30	481	8.2	1239	9.7	
31–40	227	3.9	671	5.3	
40<	380	6.4	909	7.1	
Missing	113	1.9	214	1.7	
Alcohol (drinks/week)					
None	3126	53.0	6582	51.5	0.12
< One/month	1055	17.9	2453	19.2	
< One/week	393	6.7	745	5.8	
≥ One/week	655	11.1	1822	14.3	
Missing	672	11.3	1178	9.2	
Physical activity					
None	3172	53.8	6830	53.4	0.04
Light-moderate	1257	21.3	2887	22.6	
Vigorous	1370	23.2	2863	22.4	
Missing	102	1.7	200	1.6	

*SD* standardized mean difference, *ALT* alanine transaminase, *GGT* gamma-glutamyl transferase, *BMI* body mass index, *FPG* fasting plasma glucose.

### Incidence and risk of PC among patients with pancreatitis

**Figure 1:**
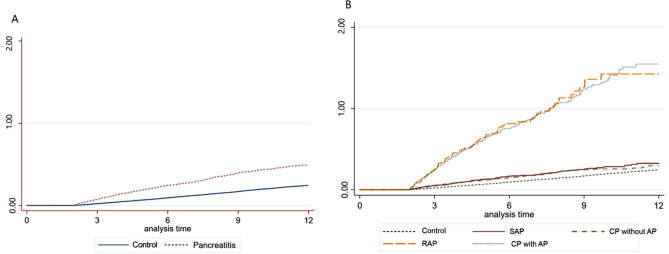
Cumulative incidences of pancreatic cancer among patients with pancreatitis followed for more than 2 years and controls. (**A**) Comparison between pancreatitis and control groups. (**B**) Comparison among SAP, RAP, CP with AP, CP without AP and control groups. SAP, single episode of acute pancreatitis; RAP, recurrent acute pancreatitis; AP, acute pancreatitis; CP, chronic pancreatitis.

The cumulative incidences of PC were compared between patients with pancreatitis and controls who were followed for more than 2 years (Fig. 1A). At 10 years, the cumulative incidences of PC were 0.43% among patients with pancreatitis and 0.20% among controls. When considering different subgroups of pancreatitis, patients with RAP and CP with AP had higher 10-year cumulative incidences of PC (1.43% and 1.37%, respectively) compared to those with SAP (0.29%) and CP without AP (0.26%) (Fig. 1B).

The incidence rate ratio (IRR) and adjusted hazard ratios (aHRs) of PC were calculated for patients with pancreatitis who were followed for more than 2 years or 5 years (Table 3). Among patients followed for more than 2 years, CP with AP had the highest risk of PC (aHR 5.74, 95% CI 5.02–6.56), followed by RAP (aHR 5.00, 95% CI 3.95–6.33), CP without AP (aHR 1.57, 95% CI 1.36–1.81), and SAP (aHR 1.32, 95% CI 1.14–1.52). Among patients followed for more than 5 years, CP with AP (aHR 4.05, 95% CI 3.25–5.04) and RAP (aHR 3.29, 95% CI 2.21–5.04) still had higher risks of PC compared to controls. The risk of PC in patients with CP without AP (aHR 1.21, 95% CI 0.96–1.53) or SAP (aHR 0.95, 95% CI 0.74–1.21) reduced to the level of controls.

**Table 3:** Incidences and risks of pancreatic cancer among patients who followed more than 2 years or 5 years after initial diagnosis of pancreatitis.

	Person-years	PC, N	Incidence/10^4^	IRR	95% CI		HR^1^	95% Cl		HR^2^	95% Cl	
> 2 years												
Control	23,883,421	3677	1.54	1.00								
SAP	900,835	208	2.31	1.50	1.30	1.73	1.64	1.42	1.88	1.32	1.14	1.52
RAP	60,673	77	12.69	8.24	6.49	10.33	6.97	5.55	8.74	5.00	3.95	6.33
CP with AP	224,402	272	12.12	7.87	6.94	8.91	7.67	6.78	8.68	5.74	5.02	6.56
CP without AP	906,143	207	2.28	1.48	1.28	1.71	1.73	1.51	1.99	1.57	1.36	1.81
> 5 years												
Control	23,876,824	1736	0.73	1.00			1.00			1.00		
SAP	900,402	72	0.80	1.10	0.86	1.39	1.25	0.99	1.59	0.95	0.74	1.21
RAP	60,510	26	4.30	5.91	3.85	8.69	5.00	3.39	7.36	3.29	2.21	4.91
CP with AP	223,840	98	4.38	6.02	4.86	7.38	5.80	4.73	7.11	4.05	3.25	5.04
CP without AP	905,721	75	0.83	1.14	0.89	1.44	1.37	1.09	1.73	1.21	0.96	1.53

*SAP* single episode of acute pancreatitis, *RAP* recurrent acute pancreatitis, *CP* chronic pancreatitis, *PC* pancreatic cancer, *IRR* incidence rate ratio, *HR* hazard ratio, *CI* confidence interval, *HR*^*1*^ adjusted sex and birth year, *HR*^*2*^ adjusted sex, birth year, hepatic fibrosis/cirrhosis, alcoholic liver disease, gallstones, ALT, GGT, BMI, FPG, cholesterol, physical activity, smoking, and alcohol drinking.

**Figure 2:**
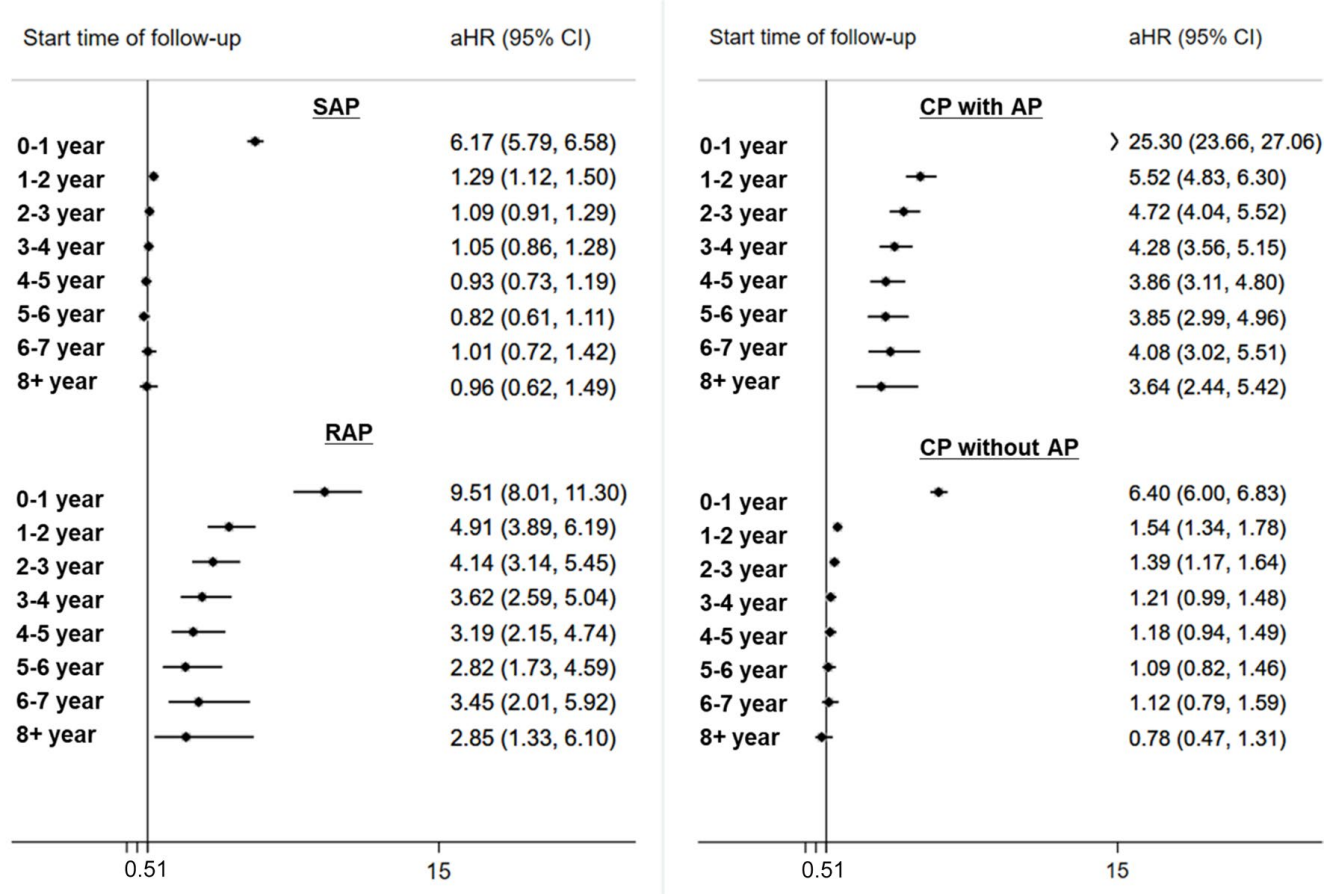
Annual risks of pancreatic cancer in patients with acute or chronic pancreatitis with time lapse after study entry. SAP, single episode of acute pancreatitis; RAP, recurrent acute pancreatitis; AP, acute pancreatitis; CP, chronic pancreatitis.

The annual risks of PC over time were analyzed to assess the risk change of PC with time lapse (Fig. 2). The majority of PC cases (94.04%) were diagnosed within 2 years of pancreatitis diagnosis. The risk of PC decreased over time, but the relative risk order among the four groups (CP with AP > RAP > CP without AP > SAP) was maintained. Higher risks of PC were observed for more than 8 years in the CP with AP or RAP groups. However, the risk of PC in patients with CP without AP or SAP decreased after 4 or 3 years, respectively.

The risks associated with various confounding factors were evaluated among patients followed for more than 2 years or 5 years (Table 4). Male, birth year, hepatic fibrosis/cirrhosis, gallstone diseases, gamma-glutamyl transferase (GGT), overweight, elevated fasting plasma glucose (FPG), and smoking were associated with a higher risk of PC (aHRs ranging from 1.16 to 1.60). However, alcohol consumption, alcoholic liver disease, physical inactivity, and cholesterol level were not significantly associated with the risk of PC. These findings were consistent for patients followed for more than 2 years or 5 years.

**Table 4:** Cox proportional hazard analysis of risk factors associated with pancreatic cancer incidences among patients followed more than 2 years or 5 years.

Parameters	> 2 Years			> 5 Years		
	HR	95% CI		HR	95% CI	
Sex (female vs. male)	1.16	1.08	1.25	1.20	1.08	1.35
Birth year	1.06	1.06	1.06	1.06	1.05	1.06
Hepatic fibrosis/cirrhosis						
No	1.00			1.00		
Yes	2.07	1.75	2.45	2.04	1.59	2.63
Alcoholic liver disease						
No	1.00			1.00		
Yes	1.04	0.87	1.23	0.94	0.73	1.21
Gallstones						
No	1.00			1.00		
Yes	1.60	1.43	1.79	1.72	1.47	2.02
ALT (U/L)						
< 40	1.00			1.00		
41+	1.00	0.89	1.12	0.85	0.71	1.01
GGT (U/L)						
< 76	1.00			1.00		
76+	1.10	1.00	1.20	1.17	1.03	1.33
BMI (kg/m^2^)						
< 18.5	1.00			1.00		
18.5–24.9	1.16	0.96	1.41	1.23	0.90	1.67
25.0–29.9	1.23	1.01	1.50	1.39	1.02	1.91
30+	1.20	0.93	1.55	1.27	0.85	1.89
FPG (mg/dL)						
< 110	1.00			1.00		
110–125	1.20	1.10	1.31	1.21	1.06	1.38
126+	1.43	1.31	1.56	1.34	1.17	1.53
Cholesterol (mg/dL)						
< 200	1.00			1.00		
200–239	0.98	0.91	1.04	0.96	0.87	1.06
≥ 240	0.99	0.91	1.09	1.08	0.95	1.23
Physical activity						
None	1.00			1.00		
Light-moderate	1.11	1.03	1.20	1.11	0.99	1.24
Vigorous	1.10	1.02	1.18	1.06	0.95	1.18
Smoking (pack-year)						
None	1.00			1.00		
< 10	1.21	1.04	1.41	1.03	0.79	1.33
11–20	1.17	1.05	1.31	1.14	0.97	1.34
21–30	1.43	1.27	1.60	1.35	1.13	1.60
31–40	1.60	1.36	1.88	1.56	1.19	2.06
40<	1.60	1.40	1.83	1.43	1.15	1.80
Alcohol drinking						
None	1.00			1.00		
< One/month	0.92	0.84	1.01	0.89	0.78	1.02
< One/week	0.90	0.80	1.01	0.91	0.77	1.07
≥ One/week	0.96	0.86	1.07	0.96	0.81	1.14

*HR* hazard ratio, *CI* confidence interval, *ALT* alanine transaminase, *GGT* gamma-glutamyl transferase, *BMI* body mass index, *FPG* fasting plasma glucose.

### Risk difference of PC between males and females

A subgroup analysis was conducted to compare the risk of PC between males and females among patients who were followed for more than 2 years. The demographic characteristics of males and females were presented (Supplement 1). Females had lower frequencies of hepatic fibrosis/cirrhosis, alcoholic liver disease, hepatic dysfunction, diabetes, alcohol drinking, and smoking compared to males. The prevalence of gallstone disease was similar between the sexes.

In terms of the IRR and aHR of PC, females exhibited higher risks compared to males in the subgroups of SAP, RAP, and CP with AP, with percentages of 37.8% in RAP, 6.2% in SAP, and 3.7% in CP with AP groups (Table 5). The aHRs of individual confounding factors were compared between males and females who were followed for more than 2 years (Supplement 2). Compared to males, females had a higher risk of PC in patients with hepatic fibrosis/cirrhosis or gallstone diseases and a lower risk of PC in patients with diabetes and smoking.

**Table 5:** Incidences and risks of pancreatic cancer in males and females who followed more than 2 years after initial diagnosis of pancreatitis.

	Person-years	PC	Incidence/10^4^	IRR	95% CI		HR	95% CI	
Male									
Control	13,683,772	2,282	1.67	1.74	1.00				
SAP	494,079	122	2.47	2.95	1.48	1.22	1.29	1.07	1.56
RAP	38,167	44	11.53	15.49	6.91	5.01	4.48	3.29	6.1
CP with AP	150,963	188	12.45	14.37	7.47	6.40	5.71	4.86	6.72
CP without AP	491,372	128	2.60	3.10	1.56	1.30	1.59	1.32	1.90
Female									
Control	10,199,649	1,395	1.37	1.00			1.00		
SAP	406,756	86	2.11	1.55	1.23	1.92	1.37	1.09	1.72
RAP	22,507	33	14.66	10.72	7.35	15.13	6.17	4.28	8.89
CP with AP	73,439	84	11.44	8.36	6.63	10.43	5.92	4.67	7.49
CP without AP	414,771	79	1.90	1.39	1.10	1.75	1.54	1.23	1.94

*SAP* single episode of acute pancreatitis, *RAP* recurrent acute pancreatitis, *CP* chronic pancreatitis, *PC* pancreatic cancer, *IRR* incidence rate ratio, *HR* hazard ratio, *CI* confidence interval, *HR* adjusted sex, birth year, hepatic fibrosis/cirrhosis, alcoholic liver disease, gallstones, ALT, GGT, BMI, FPG, cholesterol, physical activity, smoking, and alcohol drinking.

### Mortality of PC in patients with pancreatitis and controls

PC cases among patients with pancreatitis had better overall survival rates compared to controls (Table 6). The aHRs of mortality for different subgroups of pancreatitis were all lower than 1, indicating a lower risk of mortality in PC cases among the pancreatitis group. This trend was consistent in patients who followed more than 2 years or 5 years.

**Table 6:** Mortality rates of pancreatic cancer according to pancreatitis subgroups among patients who followed more than 2 years or 5 years.

	> 2 Years			> 5 years		
Groups	HR	95% CI		HR	95% CI	
Controls	1.00			1.00		
SAP	0.83	0.77	0.90	0.81	0.75	0.88
RAP	0.56	0.45	0.70	0.58	0.46	0.73
CP with AP	0.64	0.60	0.69	0.64	0.59	0.69
CP without AP	0.71	0.66	0.77	0.70	0.64	0.76

*SAP* single episode of acute pancreatitis, *RAP* recurrent acute pancreatitis, *CP* chronic pancreatitis.

## Discussion

This study demonstrated that patients with AP or CP have a higher risk of developing PC compared to the control group. Among the subgroups of pancreatitis, RAP and CP with AP groups showed a higher and more prolonged risk of PC compared to SAP and CP without AP groups. Based on these findings, we suggest that the strategy of cancer surveillance should be individualized based on the specific subgroups of pancreatitis.

The increased risk of PC has been reported in patients with CP with significant variation^3–9,18–21^. In Lowenfels et al.’s^6^ multinational study, the cumulative incidence of PC in CP patients was 1.8% at 10 years. A European cohort study revealed an incidence of PC in CP patients of 0.2% per year^18^. A study on veterans in the USA revealed the cumulative incidence of 1.04% in the CP group, which was higher than the 0.20% in the control group^3^. Recent studies conducted in South Korea reported the cumulative PC incidences of 2.20% in a hospital based study^22^ and 0.68% in a population based study^23^. Another study on patients who underwent surgery for CP showed a higher cumulative incidence of PC with rates of 1.48% at 3 years, 2.63% at 6 years, and 3.71% at 9 years after surgery^2^.

In this study, the cumulative incidences of PC at 10 years were strikingly different in the four subgroups of pancreatitis. They were 1.42% in the RAP group and 1.37% in the CP with AP group, which were compatible or slightly lower than previous results. However, they were very low in the CP without AP group (0.26%) and SAP group (0.29%), which were slightly higher than the control group (0.20%).

In this study, the risk of PC was found to be higher in patients with CP with AP compared to those with CP without AP. CP with AP has been reported to exhibit a higher symptom burden and a greater number of flares than CP without AP^12^. These patients are often active smokers and have alcohol-related etiology for their CP. The increased risk of PC in these individuals could be attributed to the “injury–inflammation–cancer” pathway^24^. Recurrent pancreatic injuries lead to a pro-inflammatory environment characterized by various immune cells, cytokines, chemokines, growth factors, and altered extracellular matrix, thereby promoting prolonged inflammatory and chronic conditions. Inflammation and oncogenes collaborate as key promoters of these diseases^25^.

Another plausible mechanism is the alcohol related diseases as the primary cause of CP with AP. Previous study has observed an independent association between inflammatory complications and alcoholic etiology^14^. However, several studies focusing on AP or CP have reported that the risk of PC does not differ based on alcoholic or non-alcoholic pancreatitis^6,11,20^. Furthermore, the higher proportion of smokers in our CP with AP cohort aligns with previous findings that most alcohol users are concurrent smokers^26^. It is important to note that smoking is a risk factor for both CP and PC.

The association between AP and PC remains a topic of controversy^2^. While some studies have reported a positive association between AP and PC^7,8,11,27^, others have found weak or no relation^2^. In this study, the risk of PC in SAP group increased by 32% compared to the control group, and this increase persisted for the first 3 years. These findings are consistent with a recent study conducted using a large veterans cohort^11^.

Regarding RAP, the magnitude of PC risk is still debated. Some studies have reported a 2.4-fold increased risk of PC in RAP compared to SAP^7^, while others have not identified a significant difference in PC risk among different subgroups of pancreatitis^8^. RAP is considered an intermediate stage in the pathogenesis of CP, and a subset of RAP patients may transit to CP during the natural course of the disease^28^. In this study, the shared clinical characteristics of patients with RAP or CP preceding AP, such as higher prevalence of alcohol consumption or smoking, are consistent to a previous study^12^. The risk of PC in the RAP group was 4.5-fold higher compared to the control group, while in the SAP group, it was a 1.32-fold higher than the control group. These findings suggest that both RAP and CP with AP are part of the same disease spectrum. These findings are consistent with a recent study reported that PC risks are related with the number of recurrence in RAP and CP with AP^11^. A recent study conducted in South Korea also reported that CP patients with parenchymal calcification have a low risk of PC^22^. Based on these findings, including our own results, it is suggested that PC risks are determined by inflammation in the pancreas rather than chronicity.

The risk of PC in patients with CP has been found to decline over time but remains persistent more than 10 years, as observed previous studies, including this one^7,8,10,11,29^. In SAP, the risk of PC disappears after 3 years, and in CP without AP, it disappears after 4 years. However, in RAP group and CP with AP group, the increased risk persists for more than 8 years. Based on these findings, it is suggested that the duration of cancer surveillance should be individualized by the specific subgroups of pancreatitis.

Previous studies have reported conflicting findings regarding the risk of PC in AP or CP between males and females^7,10,20,30^. A recent study utilizing the UK Biobank cohort reported that males with AP had a higher risk of PC than females, while females with CP had a higher risk of PC than males^29^. However, in this study, females showed a higher risk of PC in RAP, CP with AP, and SAP groups, and similar risk in CP without AP group compared to males. Although the exact reasons are not clearly defined, it is possible that males with pancreatitis exhibit more frequent additional risk factors for PC, such as hepatic fibrosis/cirrhosis, alcoholic liver disease, gallstones, hepatic dysfunction, diabetes, smoking, and alcohol drinking. Consequently, the impacts of pancreatitis on the development of PC might be lower in males compared to females.

In this study, a 2-year lag time was applied to account for the possibilities of misdiagnosis of PC as pancreatitis. Because many patients with CP are asymptomatic, they often visit the hospital due to abdominal pain, which can be associated with PC. The risk of misdiagnosis is high due to the similarities in clinical, radiological, and biochemical nature of these diseases^31,32^. To address this issue, most studies have utilized a lag time period of 1 or 2 year, as the short-term association between CP and PC risk is most likely due to the initial misdiagnosis of PC as CP^31^. However, it is important to note that applying a lag time period carries the risk of excluding a significant number of PC cases from the study. In this study, for example, 96% of PC cases were excluded by the 2-year lag period. The determination of the appropriate lag time is closely related to the accuracy of the differential diagnostic between PC and CP. Therefore, a close follow-up should be conducted in the first two years following a CP diagnosis to avoid overlooking a potential PC cases.

In this study, PC cases diagnosed in pancreatitis group were found to be young and had a better prognosis compared to those in the control group. These findings are consistent with previous studies that found patients with a recent AP were diagnosed with PC at younger age and trended to be diagnosed at an earlier stage compared to PC patients without AP^3,27^. The implementation of surveillance among pancreatitis patients may contribute to the earlier detection of PC, which in turn leads to better prognosis. However, it is important to conduct further research to validate these findings and to identify other potential factors that may contribute to the improved survival outcome in PC cases among patients with pancreatitis.

In this study, several risk factors associated with PC were adjusted for analysis. Among patients with pancreatitis, smoking was found to be a significant risk factor for PC having a 1.17–1.60-fold increased risk of PC, showing a dose–response correlation. These findings are consistent to previous research^33,34^. Smoking is a known risk factor for PC as well as for disease progression in CP^35^. Furthermore, this study revealed that overweight, prediabetes or diabetes, gallstone disease, and hepatic fibrosis/cirrhosis were associated with an increased risk of PC among patients with pancreatitis, which is in line with a previous study^36^. However, alcohol consumption was not identified as a risk factor of PC in this study or in previous studies, despite it being a known risk factor for pancreatitis and a common characteristics of patients with RAP and CP with AP^3,6^. On the other hand, physical inactivity and high cholesterol levels did not show an association with the risk of PC in this study.

The proportion of CP cases preceding AP can vary among different study populations. In our study, patients with CP who had preceding AP accounted for only one-fifths of CP cases. This proportion differs from previous studies that reported proportions of 40%^11^ or 60%^12^. Additionally, in our study, CP without AP was diagnosed at a younger age compared to CP with AP. This finding contrasts with previous result that showed a younger age in CP with AP^12^. Our data provide reliable information regarding the similar age at initial AP diagnosis in different subgroups (such as SAP, RAP, CP with AP). These inconsistent results among studies may be attributed to the differences in the study population evaluated.

The strength of this study are as follows. Firstly, we adjusted for most lifestyle or metabolic factors known to be associated with PC, such as smoking, alcohol consumption, obesity, and diabetes^37^. Additionally, we controlled for hepatic dysfunction, alcoholic liver disease, hepatic fibrosis/cirrhosis, and gallstone diseases. This adjustment helps to account for potential confounding variables and strengthens the validity of our findings. Secondly, our study included a large sample size and had a sufficient duration of follow-up. The large cohort provides robust statistical power and increases the generalizability of our results. Moreover, the adequate follow-up duration allows us to assess the long-term risk of PC in different subgroups of pancreatitis.

However, this study also has certain limitations. Firstly, we were unable to identify other potential confounding factors associated with PC, such as a family history of the disease. This missing information may introduce some degree of confounding bias into our results. Secondly, we acknowledge that the survey responses relied on the participants' memories, which could introduce recall bias. Inaccurate recall of smoking duration, intensity, and cessation may affect the accuracy of our smoking-related risk estimates.

In conclusion, this study provides valuable insights into the magnitude and duration of PC risk among different subgroups of pancreatitis. We suggest that an optimized strategy for PC surveillance should be implemented, considering the specific subgroups of pancreatitis. To improve the prognosis of PC, it is crucial to identify patients who are suitable candidates for surveillance, particularly those with RAP or CP with AP and other risk factors for PC.

## Methods

### Data source

This population-based retrospective cohort study utilized two databases: the Korean National Health Insurance (KNHI) and the National Health Screening Program (NHSP). The NHIS database contains health information from 50 million Koreans and is a mandatory health insurance program that covers 97.1% of the Korean population. It includes comprehensive data on medical services provided to patients, such as diagnoses, demographics, prescriptions, surgeries, tests, and imaging studies. The diagnoses are documented using the International Classification of Diseases (ICD)-10.

In 2005, the Korean government introduced the Support for Serious Illness program, which offered reduced coinsurance rates for registered cancer patients. To be registered in the program, patients had to receive a physician's diagnosis, which required confirmation through at least one of the following: pathology, typical radiologic findings, and/or laboratory data. The data from the Support for Serious Illness program were integrated into the KNHI database.

The NHSP recommends that insurance subscribers undergo general health screenings at least every two years. During these health examinations, patient demographics, previous medical histories, and laboratory tests are recorded. The present study utilized the NHSP-KNHI cohort.

### Study population

**Figure 3:**
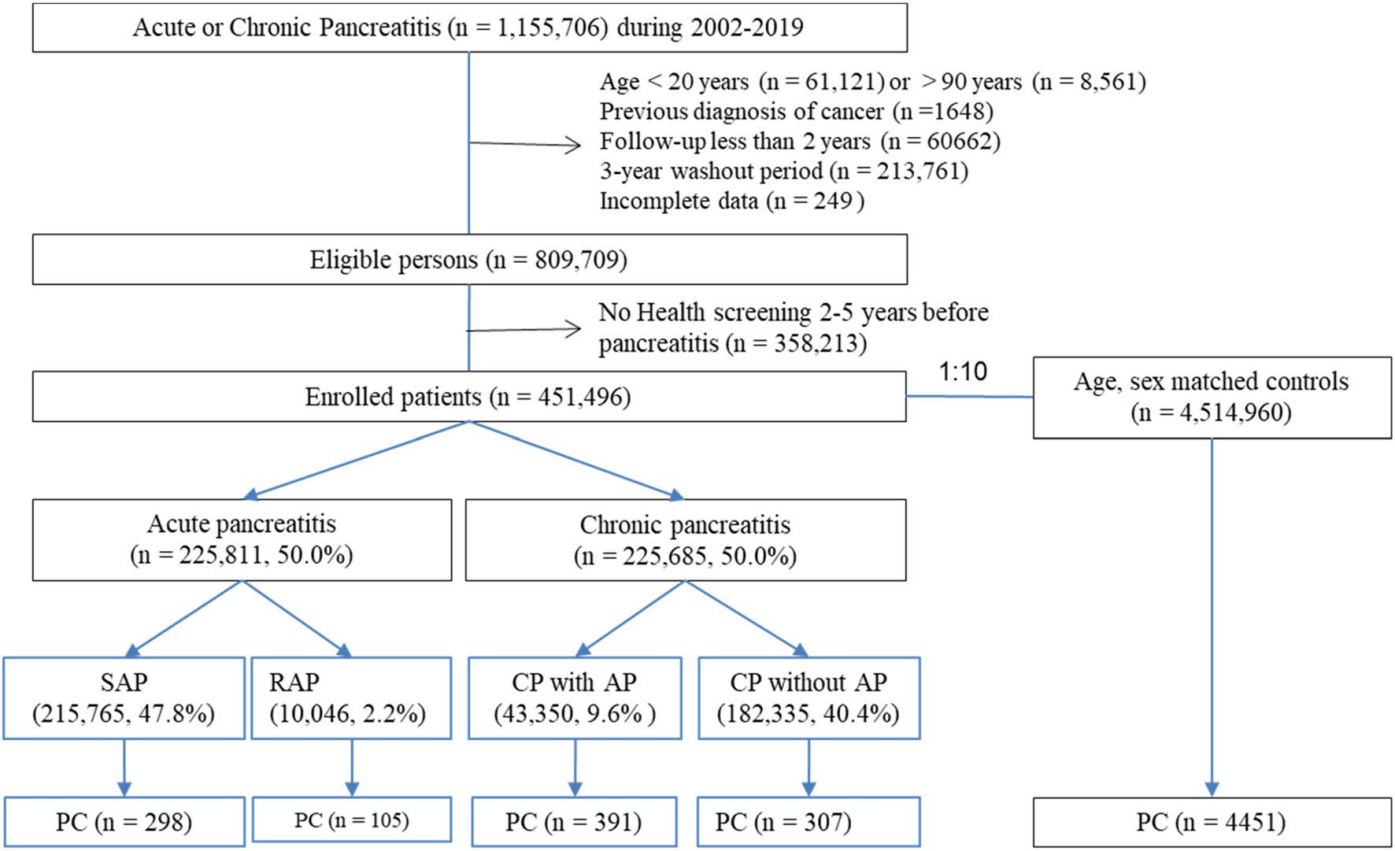
Flow chart of enrolled study populations. SAP, single episode of acute pancreatitis; RAP, recurrent acute pancreatitis; CP, chronic pancreatitis; PC, pancreatic cancer.

The study population included patients with pancreatitis, including AP and CP, as well as a matched control group. Several exclusion criteria were applied: patients younger than 20 years old (n = 61,121), patients older than 90 years old (n = 8561), patients with a previous cancer diagnosis (n = 1648), patients with less than 2 years of follow-up (n = 60,642), patients with a 3-year washout period (n = 213,761), and patients with incomplete data (n = 249). After applying these exclusions, a total of 451,496 patients and 4,514,960 matched controls were included in the study (Fig. 3).

Patients with pancreatitis were identified using the ICD-10 codes. Within the pancreatitis patient population, patients with newly diagnosed AP (inpatient, K85.x) were further classified as having a single episode (SAP) or more than two episodes (RAP). Patients with newly diagnosed CP (inpatient or outpatient, K86.0 and K86.1) were categorized into those with AP before CP diagnosis (CP with AP) and those without a history of AP before CP diagnosis (CP without AP). The study also identified patients with PC using ICD-10 codes (C25.0–25.9) and V-codes. Patients were followed up until death, PC development, or December 31, 2021, with a median follow-up time of 5.02 years (interquartile range, I25–I75; 1.94–7.14).

### Data collection and covariates

All data were obtained at the time of enrollment and from previous health-checkups conducted 2–5 years prior, utilizing the NHSP-KNHI cohort. From the NHSP data, various patient information and health parameters were obtained, including demographics, smoking status, history of cancers, alcohol consumption, body weight, height, physical activity, and levels of FPG, alanine transaminase (ALT), GGT and cholesterol.

The covariates considered in the study included demographics such as age and sex, lifestyle and risk behaviors, and disease-related variables. Lifestyle and risk behaviors encompassed smoking, drinking, and physical activity. Smoking status was categorized as none, < 10 pack-years (PY), 10–20 PY, 21–30 PY, 31–40 PY and 41+ PY. Alcohol intake was grouped as none, mild (< one/month), moderate (< one/week) and severe (≥ one/week) drinkers. Physical activity was defined as engaging in moderate-intensity physical activity for at least 30 min per day or vigorous-intensity physical activity for at least 20 min per day. It was grouped as none, light to moderate (1–4 times per week), and vigorous (≥ 5 times per week) activity.

Serum biochemical parameters, including FPG (categorized as < 100 mg/dL for normal, 100–125 mg/dL for prediabetes, and ≥ 126 mg/dL for diabetes), total cholesterol (categorized as < 200 mg/dL for normal, 200–239 mg/dL for borderline high, and ≥ 240 mg/dL for high), ALT (≤ 40 U/L for normal and + 41 U/L for high), and GGT (≤ 76 U/L for normal and + 76 U/L for high), were also included as covariates. Body mass index (BMI) was calculated as weight (kg) divided by height squared (m^2^) and categorized as < 18.5 kg/m^2^ for underweight, 18.5–24.9 kg/m^2^ for normal, 25.0–29.9 kg/m^2^ for overweight, and ≥ 30 kg/m^2^ for obesity based on the World Health Organization obesity standards. Information about alcoholic liver diseases (ICD-10 code K70.x), hepatic fibrosis/cirrhosis (ICD-10 code K74.x), or gallstone diseases (ICD-10 code K80.x) was obtained from the KNHI database.

### Statistical analysis

The statistical analysis in this study utilized Cox proportional hazard models to examine the relationships between the incidence of PC and various factors. Adjusted hazard ratios (aHRs) and 95% confidence intervals (CI) were calculated after adjusting for multiple covariates, including age, sex, smoking, alcohol consumption, obesity, levels of FPG, cholesterol, ALT, or GGT, physical activity, and diseases associated with alcohol intake or pancreatitis. Any missing data were excluded from the analysis. The standardized mean difference (SD) was used to assess the magnitude of differences between the two cohorts, with an SD ≤ 0.10 indicating a negligible difference.

The annual incidence of PC was measured as the number of cases per 10,000 individuals, with 95% CIs provided for each group. The time at risk was calculated from the date of enrollment until the date of PC diagnosis, death, or a predetermined censoring date. Vital status information for each individual was obtained from Statistics Korea. Stata/MP2 software (version 13.1; StataCorp, College Station, TX, USA) was used for all statistical analyses.

### Ethical considerations

All research processes were conducted in accordance with the relevant regulations and guidelines. This study was performed in accordance with the provisions of the Declaration of Helsinki. Ethical approval for the study was obtained from the Institutional Review Board Ethics Committee of Korea University College of Medicine (KUIRB-2021-0130-01). Informed consent was waived as the data analyses were conducted retrospectively using anonymous data obtained from the NHIS database in Korea (https://nhiss.nhis.or.kr).

### Supplementary Information

Supplementary Information 1.

## Data Availability

The datasets used and/or analyzed during the current study available from the corresponding author on reasonable request.
