# Supplementary material for: Incidence and risk of pancreatic cancer in patients with acute or chronic pancreatitis: a population-based cohort study
Source: Sci Rep. 2023 Nov 2;13:18930. doi: 10.1038/s41598-023-45382-y (PMC10622573; doi:10.1038/s41598-023-45382-y)
Supplement: Supplementary file 1 — Supplementary Information 1. [file 41598_2023_45382_MOESM1_ESM.docx]

Supplement 1. Characteristics of males and females with acute or chronic pancreatitis and controls

|  | Males | | | | | Females | | | | |
| --- | --- | --- | --- | --- | --- | --- | --- | --- | --- | --- |
|  | Control | | Pancreatitis | | SD | Control | | Pancreatitis | | SD |
| N, % | 2532180 | 100.0 | 253218 | 100.0 |  | 1982780 | 100.0 | 198278 | 100.0 |  |
| Hepatic fibrosis/cirrhosis | |  |  |  |  |  |  |  |  |  |
| No | 2463403 | 97.3 | 227788 | 90.0 | 0.30 | 1953454 | 98.5 | 189330 | 95.5 | 0.18 |
| Yes | 68777 | 2.7 | 25430 | 10.0 |  | 29326 | 1.5 | 8948 | 4.5 |  |
| Alcoholic liver disease | | | | |  |  |  |  |  |  |
| No | 2206920 | 87.2 | 181189 | 71.6 | 0.39 | 1926115 | 97.1 | 186065 | 93.8 | 0.16 |
| Yes | 325260 | 12.8 | 72029 | 28.4 |  | 56665 | 2.9 | 12213 | 6.2 |  |
| Gallstones |  |  |  |  |  |  |  |  |  |  |
| No | 2414890 | 95.4 | 209291 | 82.7 | 0.42 | 1899339 | 95.8 | 164013 | 82.7 | 0.43 |
| Yes | 117290 | 4.6 | 43927 | 17.3 |  | 83441 | 4.2 | 34265 | 17.3 |  |
| ALT (U/L) |  |  |  |  |  |  |  |  |  |  |
| < 40 | 2157805 | 85.2 | 200843 | 79.3 | 0.15 | 1872691 | 94.4 | 183329 | 92.5 | 0.08 |
| ≥ 41 | 373138 | 14.7 | 52222 | 20.6 |  | 109056 | 5.5 | 14840 | 7.5 |  |
| Missing | 1237 | 0.0 | 153 | 0.1 |  | 1033 | 0.1 | 109 | 0.1 |  |
| GGT (U/L) |  |  |  |  |  |  |  |  |  |  |
| < 76 | 2059496 | 81.3 | 178414 | 70.5 | 0.26 | 1754614 | 88.5 | 165882 | 83.7 | 0.14 |
| ≥ 76 | 471816 | 18.6 | 74687 | 29.5 |  | 227438 | 11.5 | 32311 | 16.3 |  |
| Missing | 868 | 0.0 | 117 | 0.0 |  | 728 | 0.0 | 85 | 0.0 |  |
| BMI (Kg/m^2^) |  |  |  |  |  |  |  |  |  |  |
| < 18.5 | 59934 | 2.4 | 8735 | 3.4 | 0.08 | 85146 | 4.3 | 9628 | 4.9 | 0.04 |
| 18.5–24.9 | 1534886 | 60.6 | 152783 | 60.3 |  | 1269542 | 64.0 | 124008 | 62.5 |  |
| 25.0–29.9 | 858136 | 33.9 | 82230 | 32.5 |  | 546706 | 27.6 | 55267 | 27.9 |  |
| ≥ 30 | 79224 | 3.1 | 9470 | 3.7 |  | 81386 | 4.1 | 9375 | 4.7 |  |
| FPG (mg/dl) |  |  |  |  |  |  |  |  |  |  |
| < 110 | 1977412 | 78.1 | 186337 | 73.6 | 0.11 | 1679851 | 84.7 | 163249 | 82.3 | 0.07 |
| 110–125 | 301040 | 11.9 | 33407 | 13.2 |  | 170898 | 8.6 | 18388 | 9.3 |  |
| ≥ 126 | 252870 | 10.0 | 33367 | 13.2 |  | 131311 | 6.6 | 16556 | 8.3 |  |
| Missing | 858 | 0.0 | 107 | 0.0 |  | 720 | 0.0 | 85 | 0.0 |  |
| Cholesterol (mg/dL) | |  |  |  |  |  |  |  |  |  |
| < 200 | 1497862 | 59.2 | 155609 | 61.5 | 0.05 | 1066394 | 53.8 | 109302 | 55.1 | 0.03 |
| 200–239 | 768131 | 30.3 | 70671 | 27.9 |  | 633822 | 32.0 | 61554 | 31.0 |  |
| ≥ 240 | 264961 | 10.5 | 26793 | 10.6 |  | 281563 | 14.2 | 27317 | 13.8 |  |
| Missing | 1226 | 0.0 | 145 | 0.1 |  | 1001 | 0.1 | 105 | 0.1 |  |
| Smoking (pack-year) | |  |  |  |  |  |  |  |  |  |
| None | 1014895 | 40.1 | 92195 | 36.4 | 0.11 | 1881312 | 94.9 | 184840 | 93.2 | 0.07 |
| < 10 | 363020 | 14.3 | 33887 | 13.4 |  | 60458 | 3.0 | 8095 | 4.1 |  |
| 11–20 | 456318 | 18.0 | 46550 | 18.4 |  | 19866 | 1.0 | 2671 | 1.3 |  |
| 21–30 | 305909 | 12.1 | 34003 | 13.4 |  | 6083 | 0.3 | 849 | 0.4 |  |
| 31–40 | 174049 | 6.9 | 20314 | 8.0 |  | 2376 | 0.1 | 334 | 0.2 |  |
| ≥ 40 | 174528 | 6.9 | 21685 | 8.6 |  | 1614 | 0.1 | 238 | 0.1 |  |
| Missing | 43461 | 1.7 | 4584 | 1.8 |  | 11071 | 0.6 | 1251 | 0.6 |  |
| Alcohol (drinks/week) | | |  |  |  |  |  |  |  |  |
| None | 889121 | 35.1 | 85456 | 33.7 | 0.12 | 1411541 | 71.2 | 140484 | 70.9 | 0.05 |
| < one/month | 680246 | 26.9 | 60858 | 24.0 |  | 300808 | 15.2 | 28956 | 14.6 |  |
| < one/week | 254613 | 10.1 | 24053 | 9.5 |  | 33795 | 1.7 | 3589 | 1.8 |  |
| ≥ one/week | 559730 | 22.1 | 68895 | 27.2 |  | 62831 | 3.2 | 8080 | 4.1 |  |
| Missing | 148470 | 5.9 | 13956 | 5.5 |  | 173805 | 8.8 | 17169 | 8.7 |  |
| Physical activity |  |  |  |  |  |  |  |  |  |  |
| None | 1170563 | 46.2 | 122733 | 48.5 | 0.05 | 1159735 | 58.5 | 117544 | 59.3 | 0.02 |
| Light-moderate | 773705 | 30.6 | 73646 | 29.1 |  | 430898 | 21.7 | 42642 | 21.5 |  |
| Vigorous | 559442 | 22.1 | 53887 | 21.3 |  | 363581 | 18.3 | 35251 | 17.8 |  |
| Missing | 28470 | 1.1 | 2952 | 1.2 |  | 28566 | 1.4 | 2841 | 1.4 |  |

SAP, single episode of acute pancreatitis; RAP, recurrent acute pancreatitis; CP, chronic pancreatitis; SD, standardized mean difference; ALT, alanine transaminase; GGT, gamma-glutamyl transferase; BMI, body mass index; FPG, fasting plasma glucose

Supplement 2. Cox proportional hazard analysis of risk factors associated with incidences of pancreatic cancer between males and females who followed more than 2 years

| Parameters | Males | | | Females | | |
| --- | --- | --- | --- | --- | --- | --- |
|  | HR | 95% CI | | HR | 95% CI | |
| Birth year | 1.06 | 1.06 | 1.06 | 1.06 | 1.05 | 1.06 |
| Hepatic fibrosis/cirrhosis |  |  |  |  |  |  |
| No | 1.00 |  |  | 1.00 |  |  |
| Yes | 1.94 | 1.58 | 2.38 | 2.43 | 1.81 | 3.27 |
| ALD |  |  |  |  |  |  |
| No | 1.00 |  |  | 1.00 |  |  |
| Yes | 1.03 | 0.85 | 1.24 | 1.16 | 0.77 | 1.74 |
| Gallstones |  |  |  |  |  |  |
| No | 1.00 |  |  | 1.00 |  |  |
| Yes | 1.49 | 1.30 | 1.72 | 1.78 | 1.49 | 2.14 |
| ALT (U/L) |  |  |  |  |  |  |
| <40 | 1.00 |  |  | 1.00 |  |  |
| 41+ | 1.00 | 0.87 | 1.14 | 1.02 | 0.80 | 1.29 |
| GGT (U/L) |  |  |  |  |  |  |
| <76 | 1.00 |  |  | 1.00 |  |  |
| 76+ | 1.07 | 0.96 | 1.19 | 1.16 | 1.00 | 1.35 |
| BMI (Kg/m2) |  |  |  |  |  |  |
| <18.5 | 1.00 |  |  | 1.00 |  |  |
| 18.5-24.9 | 1.14 | 0.90 | 1.45 | 1.19 | 0.87 | 1.64 |
| 25.0-29.9 | 1.17 | 0.91 | 1.50 | 1.33 | 0.96 | 1.84 |
| 30+ | 1.24 | 0.88 | 1.75 | 1.20 | 0.81 | 1.76 |
| FPG (mg/dl) |  |  |  |  |  |  |
| <110 | 1.00 |  |  | 1.00 |  |  |
| 110-125 | 1.28 | 1.15 | 1.43 | 1.06 | 0.91 | 1.24 |
| 126+ | 1.54 | 1.39 | 1.71 | 1.23 | 1.05 | 1.45 |
| Cholesterol |  |  |  |  |  |  |
| <200 | 1.00 |  |  | 1.00 |  |  |
| 200-239 | 0.95 | 0.88 | 1.04 | 1.03 | 0.92 | 1.15 |
| 240+ | 0.95 | 0.84 | 1.08 | 1.06 | 0.93 | 1.22 |
| Physical activity |  |  |  |  |  |  |
| None | 1.00 |  |  | 1.00 |  |  |
| Light-moderate | 1.11 | 1.01 | 1.22 | 1.12 | 0.98 | 1.27 |
| Vigorous | 1.09 | 0.99 | 1.19 | 1.10 | 0.97 | 1.25 |
| Smoking |  |  |  |  |  |  |
| None | 1.00 |  |  | 1.00 |  |  |
| <10 | 1.25 | 1.06 | 1.47 | 1.14 | 0.76 | 1.71 |
| 11-20 | 1.21 | 1.07 | 1.35 | 1.00 | 0.66 | 1.51 |
| 21-30 | 1.45 | 1.29 | 1.63 | 1.24 | 0.62 | 2.48 |
| 31-40 | 1.65 | 1.40 | 1.94 | 0.61 | 0.09 | 4.32 |
| 40< | 1.61 | 1.41 | 1.85 | 2.05 | 0.77 | 5.49 |
| Alcohol |  |  |  |  |  |  |
| None | 1.00 |  |  | 1.00 |  |  |
| < one/month | 0.89 | 0.80 | 0.98 | 1.00 | 0.84 | 1.19 |
| < one/week | 0.89 | 0.78 | 1.01 | 1.03 | 0.70 | 1.53 |
| ≥ one/week | 0.95 | 0.84 | 1.07 | 1.18 | 0.75 | 1.85 |

HR, hazard ratio; CI, confidence interval; ALT, alanine transaminase; GGT, gamma-glutamyl transferase; BMI, body mass index; FPG, fasting plasma glucose
